# ﻿The rediscovery of *Ohwialuteola* (Fabaceae, Papilionoideae) after 50 years and comparative analysis of *Ohwia* species in plastid genome sequence

**DOI:** 10.3897/phytokeys.253.147019

**Published:** 2025-03-07

**Authors:** Lei Peng, Yu-Jie Zhang, Yun-Yun Xiao, Chu-Yi Xia, Xin Luo, Wei-Qiang Qin, Dai-Gui Zhang, Qiang Zhou, Ze-Long Nie, Meng-Hua Zhang

**Affiliations:** 1 Key Laboratory of Plant Resources Conservation and Utilization, College of Biology and Environmental Sciences, Jishou University, Jishou 416000, Hunan, China Jishou University Jishou China; 2 Zhangjiajie Campus, Jishou University, Zhangjiajie 427000, Hunan, China Jishou University Zhangjiajie China

**Keywords:** Desmodieae, morphology, *Ohwialuteola*, phylogeny, plastome

## Abstract

*Ohwialuteola* (H. Ohashi & T. Nemoto) H. Ohashi is only known from one collection in Yunnan Province, China. It has not been recollected since its last collection in 1972. Here, we report the rediscovery of the species that means the first new record in Hunan Province, China. Based on fresh material, we present a revised morphological description of *O.luteola* and conducted sequencing and assembly of the plastid genome. Morphologically, *O.luteola* is similar to *O.caudata*, but the former can be easily distinguished by leaflets length/width ratio ranging from 2.5 to 3.6, leaflets apex acute (with an angle of 50°–80°), terminal inflorescences, wings distinctly auriculate at base and inner side indistinctly rugose, and hilum center not over 3/5 length of seed. Molecular phylogenetic analysis confirmed *O.luteola* is sister to *O.caudata*.

## ﻿Introduction

Fabaceae (or Leguminosae), the third largest family of angiosperm, comprises more than 19,500 species in ca. 765 genera, 36 tribes, and 6 currently recognized subfamilies (Caesalpinioideae, Cercidoideae, Detarioideae, Dialioideae, Duparquetioideae, and Papilionoideae) (Azani et al. 2017). The legume plants have highly diversified in growth forms including trees, shrubs or herbs, sometimes climbing or decumbent, and ca. 88% of legume species have the ability to establish associations with nitrogen-fixing bacteria (Sprent et al. 2017; Zhang et al. 2020). Many legume species are economically and ecologically important (Yahara et al. 2013).

*Ohwia* H. Ohashi, is a small genus within the tribe Desmodieae of subfamily Papilionoideae containing two species, i.e., *O.luteola* (H.Ohashi & T.Nemoto) H. Ohashi and *O.caudata* (Thunb.) Ohashi (Huang and Ohashi 2010). Members of this genus are characterized by their shrub or subshrub growth habit, featuring pinnately trifoliolate leaves, persistent stipules, and winged petioles. *Ohwialuteola* is endemic to Yunnan Province (China) and characterized by corolla pale yellow, while *O.caudata* widely distributed in East Asia, and corolla greenish or yellowish white (Huang and Ohashi 2010). *Ohwialuteola* was described in 1998 based on a single number collection from northeastern Yunnan Province (China) in 1972 (Ohashi and Nemoto 1998), but additional specimens of *O.luteola* have not been recorded for more than 50 years.

In a recent exploration of Zhangjiajie city (Hunan Province, China), we collected an unknown *Ohwia* species with similar morphological characteristics to *O.caudata*. However, they have smaller leaflets with obtuse apex, which are obviously different from *O.caudata*. After having a determination of the material by Hiroyoshi Ohashi, one of the original authors of *Ohwialuteola* as *Desmodiumluteolum* H.Ohashi & T.Nemoto, we made a morphological comparison of our material with the images of the type of *O.luteola* (KUN) and habitat description and confirmed that they belong to *O.luteola*. Therefore, the purpose of our research described here was to provide an insight into the taxonomic status of the *O.luteola* by comparing morphological features and analyzing the plastome.

## ﻿Material and methods

### ﻿Morphology observation and measurement

In total, 9 individuals of the *O.luteola* and 6 individuals of *O.caudata* were examined and herbarium voucher specimens deposited in the herbariums of the Department of Biology, Jishou University (JIU) and the Kunming Institute of Botany (KUN). Fourteen morphological characters were selected for the morphometric analysis. The characters include terminal leaflet length; terminal leaflet width; lateral leaflet length; lateral leaflet width; petiole width; terminal leaflet petiole length; lateral leaflet petiole length; number of inflorescences per branch; number of flower nodes per inflorescence; flower stipe length; wing base (1-slightly auriculate, 2-distinctly auriculate); terminal leaflet length/width ratio; lateral leaflet length/width ratio. We performed a principal component analysis (PCA) using R v.4.0.2 (R Core Team 2020) to project and visualize trends in morphological variability across our samples.

### ﻿DNA extraction and sequencing

Total genomic DNA was extracted from silica gel-dried materials and herbarium material (three individuals of *O.luteola* and one individual of *O.caudata*) using the Plant Genomic DNA Kit (TianGen Biotech, Beijing, China) following the manufacturer’s protocol. DNA libraries were constructed with paired-end reads (PE150) were generated using an Illumina NovaSeq 6000 platform. Library construction and sequencing were carried out at Novogene Co., Ltd. in Beijing, China. Approximate 4 Gb of raw-reads were obtained for each sample.

### ﻿Plastid genome assembly, annotation, and comparison

Plastomes were assembled using GetOrganelle (Jin et al. 2020) based on the clean reads. The plastome of *O.caudata* (MG867572) was selected as a reference (Jin et al. 2019). We detected the boundaries of large single-copy (LSC), small single-copy (SSC), and two inverted repeats (IRs) using RepeatFinder v.1.0.1 (Volfovsky et al. 2001). The final annotation was conducted in GENEIOUS v. 11.1.4 (Kearse et al. 2012). A circular plastome map was drawn in OGDraw v.1.3.1 (Greiner et al. 2019). SSRs are tandem repeats of one to six nucleotide long DNA motifs with high variability, multi-allelic nature, codominant inheritance, repeatability, relative abundance, and other traits that hold great promise in evolutionary and population genetics studies. The MISA program (http://pgrc.ipk-gatersleben.de/misa/) was used to identify the SSR, with a minimum number of repeat units of 10, 5, 4, 3, 3, and 3 for mono-, di-, tri-, tetra-, penta-, and hexa-nucleotides, respectively.

### ﻿Phylogenetic analyses

We failed to obtain a complete plastid genome of the sample of ‘*O.caudata* 928’ (isotype) because the DNA of this sample was extracted from herbarium material collected over 50 years ago. To determine the phylogenetic position of *O.caudata*, a total of 34 plastid CDS were extracted using GENEIOUS v.11.1.4. The outgroups and other Leguminosae species were selected based on the work of Jin et al. (2019). Voucher information and GenBank accession numbers were provided in Appendix 1. Sequences were aligned with MAFFT (Katoh and Standley 2013). The concatenated plastid CDS dataset is deposited in DRYAD (https://doi.org/10.5061/dryad.4qrfj6qn5). Maximum likelihood (ML) analysis was performed using RAxML-HPC v.8.2.4 (Stamatakis 2014), with the GTR + I + G model and run for 1000 bootstrap iterations. The phylogenetic trees were visualized using FigTree v.1.4.2 (Rambaut 2014).

## ﻿Results and discussion

**Figure 1.**
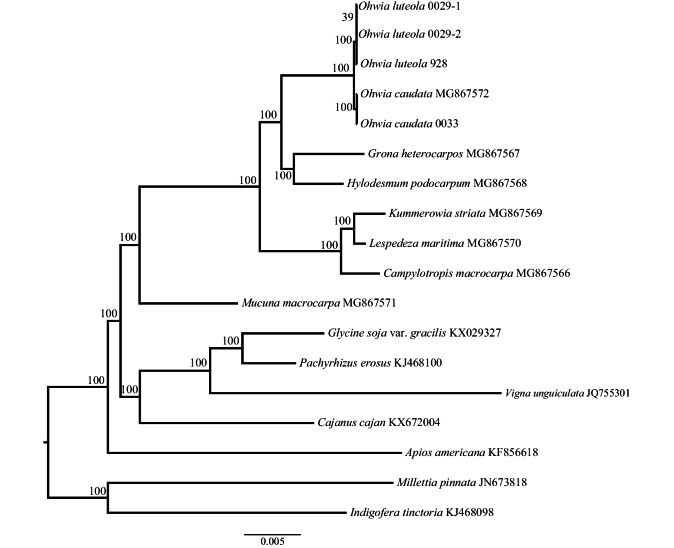
Maximum likelihood (ML) analysis based on the 34 plastid protein-coding genes. ML bootstrap (BS) values are given above the branches.

The aligned plastid CDS matrix contained 34,582 sites. The ML tree is shown in Fig. 1. Our results showed that *O.luteola* from Hunan Province and isotype from Yunnan Province were clustered together and strongly supported *O.luteola* sister to *O.caudata* (BS = 100%, Fig. 1). This sister relationship is also supported by morphological characters. Morphological synapomorphies of *O.luteola* and *O.caudata* included pinnately 3-foliolate, stipules persistent, calyx campanulate and 4-lobed (Huang and Ohashi 2010).

**Figure 2.**
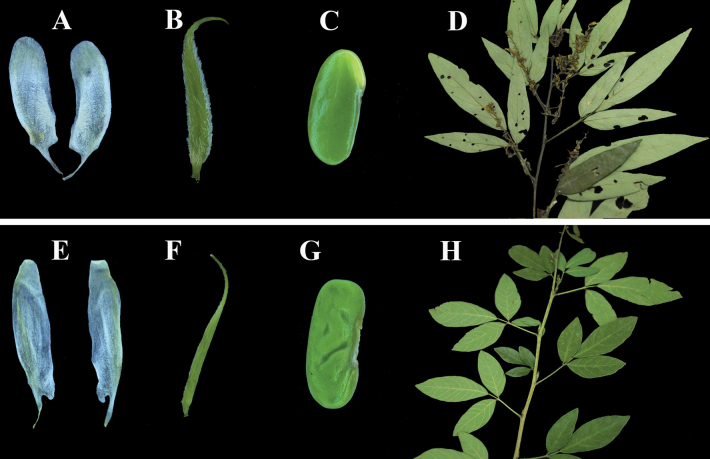
Comparison of *Ohwialuteola* and *O.caudata***A–D***O.caudata* (**A** wings **B** ovary **C** seed **D** Branch) **E–H***O.luteola* (**E** wings **F** ovary **G** seed **H** Branch).

**Figure 3.**
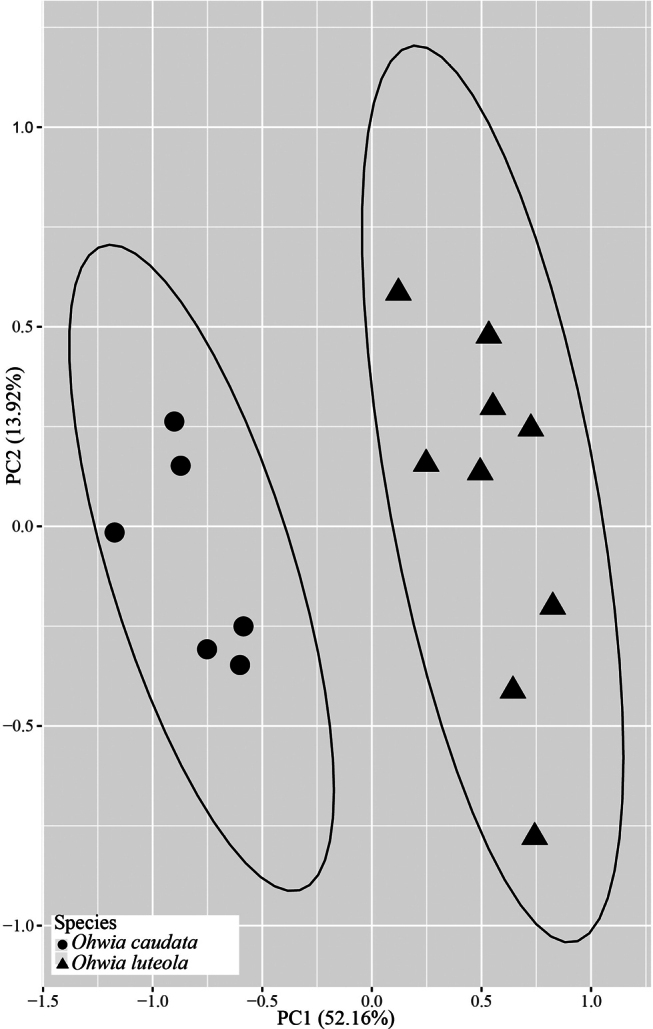
Principal components plots based on fourteen morphological characters.

Morphologically, most leaflets of *O.caudata* are lanceolate or oblong (Fig. 2D) (Ohashi 2005), terminal leaflets have a length/width ratio of up to 6.7, and leaflets apex acuminate. In contrast, *O.luteola* has oblong-elliptic leaflets, the terminal leaflet length/width ratio ranges from 2.9 to 3.6, and the leaflets apex is acute (Fig. 2H). The wings of *O.luteola* are distinctly auriculate at the base, and the inner side is indistinctly rugose (Fig. 2E) (vs. wings slightly auriculate at the base and inner side distinctly rugose). Also, it differs by its hilum at the center of the axis and not over 3/5 length of seeds (Fig. 2G) (vs. hilum off-center and over 1/2 length of seeds). More importantly, *O.luteola* grows on limestone along the river, and *O.caudata* usually grows under the forest. It is noteworthy that the corolla of *O.luteola* is described as pale yellow (Ohashi and Nemoto 1998), and the flowers observed from fresh materials collected in Hunan province are greenish-white to yellowish-white. Additionally, the flowers of the specimen turn yellow after drying. Morphological traits from 15 specimens were explored using PCA (Fig. 3). The first two principal components identified by PCA accounted for 66.08% of the variation across all characters. The PCA results showed that individuals of *O.luteola* and *O.caudata* formed distinct clusters.

**Figure 4.**
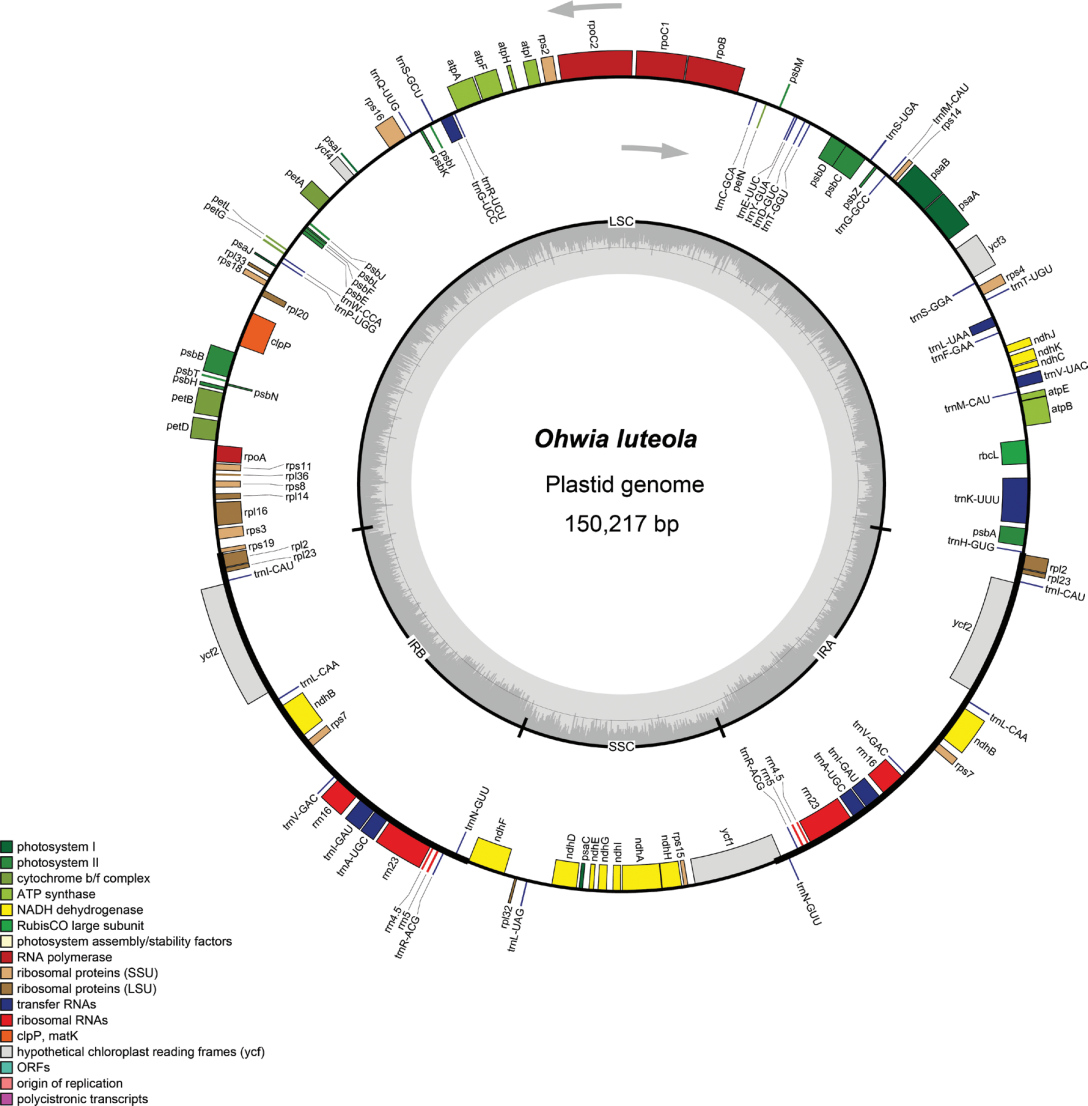
Plastid genome map of *Ohwialuteola*.

**Figure 5.**
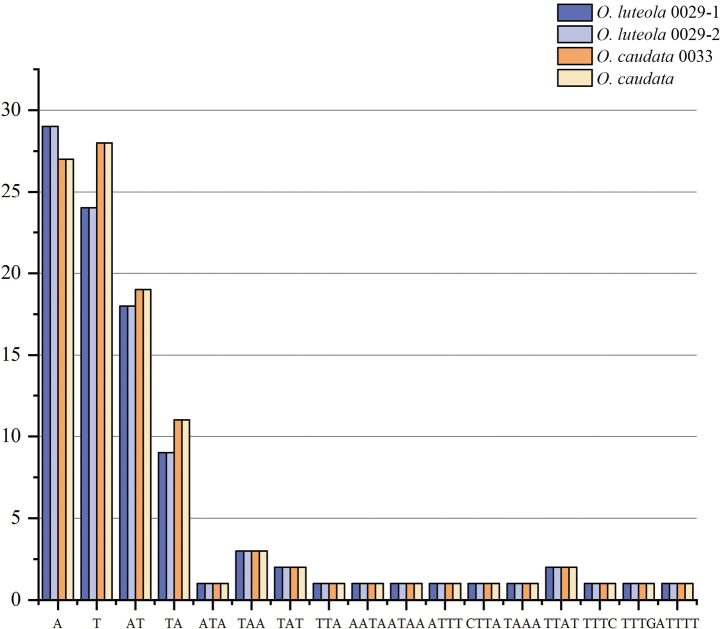
Specific forms of SSRs in 4 genomes from *Ohwia*.

We sequenced, assembled, and annotated three plastomes representing *O.luteola* (two individuals) and *O.caudata* (one individuals). The features of these plastomes are summarized in Table 1. Plastome map for the *O.luteola* is shown in Fig. 4. Consistent with previous studies in legumes, the plastomes were highly conserved, with no structural variations or content rearrangements (Jin et al., 2019). The plastome sizes of the *Ohwia* species ranged from 150,217 bp for *O.luteola* to 150,250 bp for *O.caudata*. All the two species presented a classical quadripartite structure, a LSC, an SSC, and two IRs. The length of the LSC region ranged from 83,227 bp to 83,242 bp. The SSC region varied from 18,442 bp to 18,480 bp in length, and that of the IR regions ranged from 24,264 bp to 24,274 bp (Table 1). A total of 128 genes were identified, including 83 protein-coding genes, 37 transfer RNA (tRNA) genes, and 8 ribosomal RNA (rRNA) genes. The GC content of the two species was identical in the whole chloroplast genome (35.1%), with the GC content in the IR regions (42.0%) noticeably higher than that in the SSC (28.3%) and LSC (32.6%) regions in each chloroplast genome. Our study identified a total of 384 SSRs in the two *Ohwia* species (Fig. 5). The number of SSRs in *Ohwiacaudata* is 95, while the number of simple repeats in *O.luteola* is 97. Among them, the A/T mononucleotide SSRs are the most abundant.

**Table 1.** Plastome characteristics of *Ohwialuteola* and *O.caudata*.

Species	Total size (bp)	Length of LSC (bp)	Length of SSC (bp)	Length of IRs (bp)	GC content (%)	No. of genes
*O.luteola* 0029-1	150,217	83,227	18,442	24,274	35.1%	128
*O.luteola* 0029-2	150,217	83,227	18,442	24,274	35.1%	128
*O.caudata* 0033	150,250	83,242	18,480	24,264	35.1%	128
*O.caudata*	150,249	83,241	18,480	24,264	35.1%	128

### ﻿Taxonomy

#### ﻿ Ohwia luteola

Taxon classificationPlantaeFabalesFabaceae

(H. Ohashi & T. Nemoto) H. Ohashi

282DB6A8-11E1-5AFA-8C93-8F4A73F8472F

Fig. 6

##### Diagnosis.

*Ohwialuteola* resembles *O.caudata* but differs from the latter by having terminal leaflets length/width ratio rang from 2.9 to 3.6 (vs. terminal leaflets length/width ratio rang from 4.2 to 6.7), leaflets apex acute (vs. acuminate) terminal inflorescences (vs. terminal and axillary), wings with distinctly auriculate at base, inner side indistinctly rugose (vs. wings with slightly auriculate at base, inner side distinctly rugose), hilum center, not over 3/5 length of seed (vs. hilum off-center, over 1/2 length of seed).

**Figure 6.**
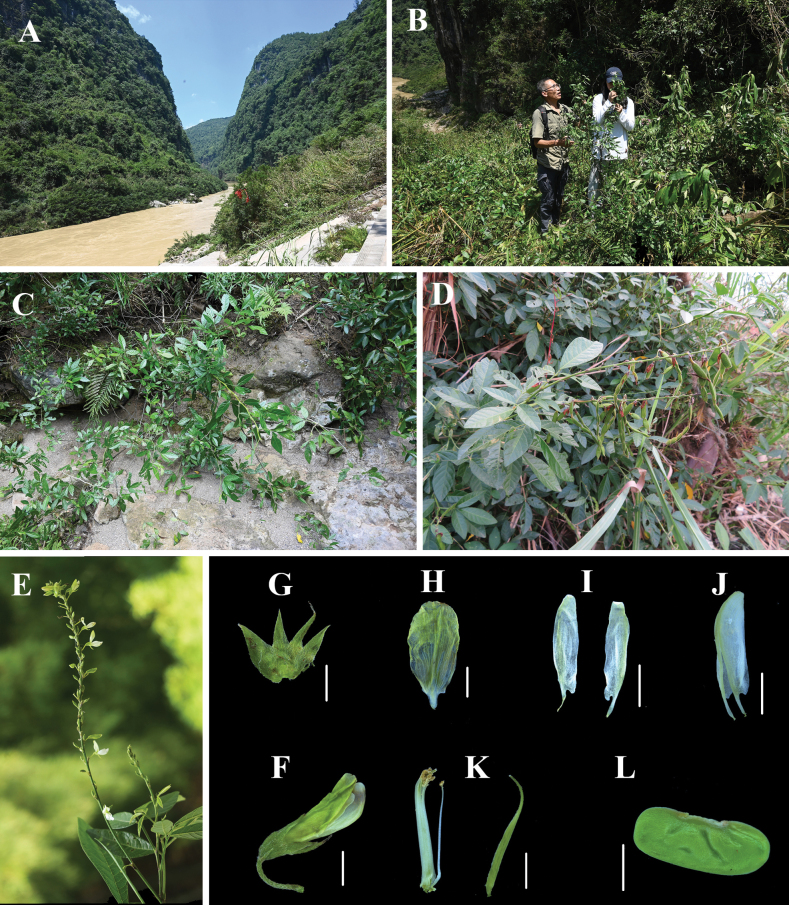
*Ohwialuteola***A** habitat **B, C** habit **D** fruits **E** inflorescence **F** flower **G** calyx **H** standard **I** wings **J** keel-petal **K** ovary and stamens **L** seed.

##### New record.

Populations of *Ohwialuteola* are known from Xixiping Street, Yaping village, and Bamaoxi village of Zhangjiajie. It is growing on limestone along the Lishui River. The companion species mainly including *Adina rubella* Hance, *Distyliumbuxifolium* (Hance) Merr., and *Cornusquinquenervis* Franch.

##### Specimens examined.

China • Hunan: Zhangjiajie City, Yongding District, Sanjiaguan Township, Yaping village, under Zhanghua Lishui Large Bridge, on limestone areas along Lishui River, alt. 218 m, 29.111375°N, 110.258679°E, 31 Aug. 2023, *M. H. Zhang et al. 0029* (JIU); • Yunnan: Jinping County, Laomeng River, alt. 750 m, 20 May 1974, *Lüchun Exped. 944* (KUN 0608532); • Yiliang County, Niujie, alt. 450 m, 23 Sep. 1972, *Northeast Yunnan Exped. 928* (KUN 0608538).

##### Revised description.

Shrubs, erect, 1–2 m tall, main stem ca. 1 cm in diam at base, much branched. Leaves 3-foliolate, thickly papery to subleathery, both surfaces pilose and more densely hairy on raised veins, margin entire. Petiole 2–3 cm long, with narrowly winged on both, 0.2–0.3 mm wide. Terminal leaflet oblong-elliptic, widest near the middle part, 4–7.1 × 1.5–2.4 cm, principal veins 10–14 pairs, reaching the leaf margin, apex acute, base cuneate, small petiole 0.8–1.2 cm long, pubescent. Lateral leaflets smaller, 3.7–6.4 × 1.1–1.8 cm, small stipe 0.2–0.3 cm long, widest near the middle part, principal veins 6–12 pairs, reaching the leaf margin, apex acute, base cuneate, small petioles 0.2–0.3 cm long, densely pubescent. Stipules 3–7 mm long, ca. 1.0 mm wide at the base, densely pubescent, persistent. Inflorescences terminal, 7–19 cm long, rachis densely pubescent intermixed with minute uncinate and appressed or spreading longer hairs, 2–4-flowered at each node; bracts subulate, ca. 0.3 cm long. Pedicels 0.4–0.6 cm long, densely pubescent. Calyx campanulate, 0.8–1.2 cm long, outside densely appressed pubescent, 4-lobed, lobes united for ca. 1/2 length, lobes ca. 0.5 cm long, longest one linear-lanceolate. Corolla greenish-white or yellowish-white, ca. 1.5 cm long, distinctly veined; standard elliptic, 0.8–1.7 × 0.5–1.0 cm, claw ca. 2.5 mm, slightly auriculate at base, apex slightly retuse; wings shorter than keel, 1.3–1.6 cm long, apex obtuse, lamina narrowly elliptic, distinctly auriculate at base, claw ca. 3 mm, keel 0.8–1.8 cm long, apex rounded, slightly auriculate at base, claw ca. 3 mm. Vexillary stamen slightly connate at base from other 9, ca. 1.6 cm long, puberulent at upper part; remaining 9 stamens connate for 4/5 or more of length, puberulent at upper part. Style curved upward, ovary densely ap-pressed pilose on both sutures. Disk present at base of pistil. Legume linear, flat, 3.5–7 cm long, stipe ca. 5 mm long, 3–6-jointed; articles nearly rectangle, 1–1.3 × 0.5–0.7 cm, with dense, transparent to brown, uncinate hairs. Seeds compressed, reniform, ca. 12 × 5 mm; hilum center, not over 3/5 length of seed. Flowering from July to early September; fruiting from September to November.

##### Conservation status.

During our field investigations in 2022 and 2024, many populations of *O.luteola* were found in Zhangjiajie. The number of individuals of each population ranges from tens to hundreds. In addition, it is distributed along the river. We believe that it should have a much wider distribution than is currently known. Due to its wide distribution range and large population size, *O.luteola* is here recommended as Least Concern (LC) (IUCN 2022).

## Supplementary Material

XML Treatment for Ohwia luteola

## ﻿Additional information

### Author ORCIDs

Lei Peng https://orcid.org/0009-0005-7893-7076

Yu-Jie Zhang https://orcid.org/0009-0007-9098-2827

Chu-Yi Xia https://orcid.org/0009-0004-4160-297X

Dai-Gui Zhang https://orcid.org/0000-0002-1587-2739

Ze-Long Nie https://orcid.org/0000-0001-8065-3981

Meng-Hua Zhang https://orcid.org/0000-0001-5326-6994

### Data availability

All of the data that support the findings of this study are available in the main text.

## ﻿Appendix 1

**Table A1.** Species sequence information downloaded from the GenBank.

Taxon	Locality	Voucher information	GenBank number
*Apiosamericana*			KF856618
*Cajanuscajan*			KX672004
*Campylotropismacrocarpa*	Mt. Hwanghak, Chilgok-gun, Gyeongsangbuk-do, Korea	109901	MG867566
*Desmodiumheterocarpon*	Sallokdoro, Seogwipo-si, Jeju-do, Korea	98555	MG867567
*Glycinegracilis*			KX029327
*Hylodesmumpodocarpum*	Mt. Geomdan, Gwangju-si, Gyeonggi-do, Korea	169505	MG867568
*Kummerowiastriata*	Mt. Geomdan, Gwangju-si, Gyeonggi-do, Korea	DP167901	MG867569
*Lespedezamaritima*	Peak Gyeokja, Bogil-myeon, Wando-gun, Jeollanam-do, Korea	DP149121	MG867570
*Mucunamacrocarpa*	Kunigami, Okinawa, Japan	15001	MG867571
*Ohwiacaudata*	Jeju-do, Korea	NIBR378625	MG867572
*Ohwiacaudata*	Zhangjiajie, Hunan	M.H. Zhang et al., 0033 (JIU)	*
*Ohwialuteola*	Zhangjiajie, Hunan	M.H. Zhang et al., 0029-1 (JIU)	*
*Ohwialuteola*	Zhangjiajie, Hunan	M.H. Zhang et al., 0029-2 (JIU)	*
*Ohwialuteola*	Yiliang County, Yunnan	Northeast Yunnan Exped. 928 (KUN).	#
*Pachyrhizuserosus*			KJ468100
*Vignaunguiculata*			JQ755301
Outgroups			
*Indigoferatinctoria*			KJ468098
*Millettiapinnata*			JN673818

An asterisk (*) indicates newly generated plastomes; a hashtag (#) indicates plastid genome assembly failed and extracted protein-coding genes can be obtained in DRYAD (https://doi.org/10.5061/dryad.4qrfj6qn5).
